# Cartilage stem/progenitor cells‐derived exosomes facilitate knee cartilage repair in a subacute osteoarthritis rat model

**DOI:** 10.1111/jcmm.18327

**Published:** 2024-04-25

**Authors:** Jing Chen, Xiaohui Ni, Jian Yang, Hongwei Yang, Xiaoyu Liu, Minhao Chen, Cheng Sun, Youhua Wang

**Affiliations:** ^1^ Department of Orthopedics Affiliated Hospital of Nantong University, Nantong University Nantong Jiangsu Province China; ^2^ Key Laboratory of Neuroregeneration of Jiangsu and Ministry of Education, Co‐innovation Center of Neuroregeneration Nantong University Nantong Jiangsu Province China; ^3^ Department of Orthopedics Dafeng People's Hospital Yancheng Jiangsu Province China; ^4^ Department of Orthopedics Affiliated Nantong Hospital 3 of Nantong University Nantong Jiangsu Province China

**Keywords:** cartilage repair, cartilage stem/progenitor cells, CDK9, chondrocytes, exosomes

## Abstract

Cartilage defects in the knee are often associated with the progression of degenerative osteoarthritis (OA), and cartilage repair is a useful strategy for managing this disease. However, cartilage repair is challenging because of the unique environment within the tissue. Recently, stem cell‐based therapies have shed new light on this issue. In this study, we prepared exosomes (EXOs) from cartilage stem/progenitor cells (CSPCs) and found that treatment with EXOs increased the viability, migration, and proliferation of cultured primary chondrocytes. In a subacute OA rat model, the application of EXOs facilitated cartilage regeneration as evidenced by histological staining. Exosomal protein analysis together with bioinformatics suggested that cyclin‐dependent kinase 9 (CDK9) is a key factor for chondrocyte growth and migration. Functional studies confirmed this prediction, that is, inhibiting CDK9 reduced the beneficial effects induced by EXOs in primary chondrocytes; while overexpression of CDK9 recapitulated the EXOs‐induced phenotypes. RNA‐Seq data showed that a set of genes involved in cell growth and migration were up‐regulated by EXOs in chondrocytes. These changes could be partially reproduced by CDK9 overexpression. Overall, our data suggest that EXOs derived from primary CSPCs hold great therapeutic potential for treating cartilage defect‐associated disorders such as degenerative OA, and that CDK9 is a key factor in this process.

## 1 INTRODUCTION

Osteoarthritis (OA) is a prevalent degenerative joint disease characterized by the progressive destruction of articular cartilage, resulting in substantial joint deterioration and synovial inflammation, consequently causing pain, functional deterioration and reduced quality of life.^1^ With the aging population and increasing prevalence of obesity, the burden of this multifaceted syndrome has increased over the past few decades.^1^, ^2^, ^3^ Cartilage injuries and diseases have long posed a formidable challenge in medicine, largely attributable to the avascular nature of cartilage, that leads to insufficient nutrient supply and a lack of cellular support within the tissue.^4^ Developing effective therapeutic strategies to halt OA progression and stimulate cartilage regeneration are of great importance in orthopaedic research.

The use of mesenchymal stem cells (MSCs) has emerged as a promising therapeutic approach for knee OA because of their capacity to differentiate into chondrocytes at the site of injury.^5^, ^6^, ^7^ However, stem cell therapies have certain limitations, including technical constraints in large‐scale expansion in vitro, low survival rates in vivo, and potential immune rejection.^8^ In recent years, extracellular vesicles including exosomes (EXOs) have emerged as promising candidates for cartilage regenerative therapy.^9^, ^10^ EXOs derived from stem/progenitor cells and their derivatives have shown therapeutic outcomes comparable to cell‐based therapies.^11^ Utilizing EXOs not only eliminates the requirement of a substantial quantity of cells but also resolves the low viability issue observed in cell therapies.^12^ Moreover, EXOs ensure safety through their efficient targeting mechanisms and rapid clearance from the body.^13^ Additionally, the therapeutic potential of MSCs has been primarily attributed to their paracrine activity, particularly involving EXOs.^14^

In this study, we examined the therapeutic effects of EXOs secreted by primary cartilage stem/progenitor cells (CSPCs) on cartilage regeneration. Our results showed that CSPC‐EXOs substantially promoted cartilage repair in a subacute OA rat model. We speculate that this was due to the improved proliferation and migration of chondrocytes. Our proteomics analysis, bioinformatics‐based predictions, and functional studies revealed that exosomally‐derived cyclin‐dependent kinase 9 (CDK9) plays a key role in cartilage regeneration. These findings highlight the considerable therapeutic potential of CSPC‐EXOs in treating cartilage dysfunction‐associated diseases, such as OA.

## 2 MATERIALS AND METHODS

### 2.1 Culture of primary cartilage stem/progenitor cells (CSPCs) and chondrocytes

The fibronectin adhesion technique was used to isolate primary CSPCs and chondrocytes from rat knee cartilages.^15^ Briefly, 6‐well plates were coated with fibronectin the day before and left overnight at 4°C. Cartilage tissue from the femoral trochlear of rat knee joints was isolated under sterile conditions, washed three times with PBS, and carefully cut into small pieces. Then, the mixture was poured into a 15 mL centrifuge tube and digested with 0.25% trypsin for 10–15 min. Next, F12 medium (containing 10% fetal bovine serum and 100 U/mL penicillin and streptomycin) was added to terminate digestion. After washing three times with PBS, 0.02% type II collagenase was added for further digestion in a shaker for 5–7 h at 37°C. The digested tissue mixture was filtered through a 40 μm filter, centrifuged at 1500 rpm for 5 min, and then resuspended in DMEM/F12 medium. Finally, the isolated CSPCs and chondrocytes were seeded into 6‐well plates and incubated at 37°C and 5% CO_2_ humidity‐controlled incubator for 18–20 min to allow cell attachment. Cells suspended in culture medium were collected and placed in a new culture dish where chondrocytes were cultured, while a monolayer of CSPCs remained at the bottom of the well. The adherent cells were passaged in F12 medium containing 10% fetal bovine serum, 100 U/mL penicillin and streptomycin at 37°C with 5% CO_2_. Subsequently, cells were passaged every 3 days, and P3–P4 cells were used for further experiments. All surgical and postoperative animal care was carried out in accordance with the Guidelines for the Care and Use of Experimental Animals (National Research Council) and were approved by the Animal Research Ethics Committee of Nantong University.

### 2.2 Immunofluorescence

P2–P3 chondrocytes were seeded on small round glass slides. After 3‐time washing in PBS, cells were fixed in 4% formaldehyde for 15 min, and then cells were treated with 0.5 Triton X‐100 for 10 min at room temperature. After washing, cells were incubated with anti‐Collagen II (Servicebio, Wuhan, China; GB11021) overnight at 4°C. The primary antibody was collected and then cells were incubated with goat anti‐rat IgG H&L (Alexa Fluor 488) for 1 h at room temperature. DAPI (Sigma‐Aldrich; D9542) was used to stain the nuclei. Cell images were taken with a fluorescence microscope (Life Technology; Evos FL Auto).

### 2.3 CSPC‐EXO isolation and identification

After cells reached 80% confluency, cell culture medium was changed to serum‐free medium and cultured for 48 h. Cell culture medium was collected and centrifuged at 500 × *g* for 10 min to remove cells; The supernatants were subjected to serial centrifugation including 3000 × *g* for 15 min to remove cell debris, and 10,000 × *g* for 30 min to remove larger vesicles, then filtered through a 0.22 μm filter (Millipore). After filtration, samples were centrifuged at 100,000 *g* for 90 min. Discard the supernatants, resuspend precipitated EXOs in the bottom of the tubes with 10 mL PBS, and centrifuge again at 100,000 *g* for 90 min. Discard the supernatants, resuspend precipitated EXOs with 200 μL PBS and store at −80°C until use.

Transmission electron microscopy (TEM; Hitachi, Japan) was used to observe EXO morphology. Zeta View 8.05.04 software (German Particle Metrix) and nanoparticle tracking analysis (NTA, Particle Metrix, Germany) were used to analyse EXO diameter and concentration. Protein concentration was measured using bicinchoninic acid assay (Pierce; 23225). Western blot analysis was employed to detect EXO markers including CD9 and CD63. Calnexin was used as a negative control.

### 2.4 EXO labeling and internalization

PKH26 (Sigma‐Aldrich; PKH26PCL) was used to label EXOs according to the manufacturer's instructions. Excess dye was removed by centrifugation at 5000 × *g* for 17 min at 4°C using Amcon Ultra‐15 tubes (Millipore; UFC9050). After washing three times in PBS, the pellets were resuspended in PBS and designated as labelled EXOs. The labelled EXOs were co‐cultured with rat primary chondrocytes at a concentration of 1 × 10^9^/mL under serum‐free conditions at 37°C for 24 and 48 h. Cells were fixed with 4% paraformaldehyde, and the nuclei were stained with DAPI. The cell skeleton was stained with FITC‐Phalloidin (Sigma‐Aldrich; P5282). Internalization of EXOs was monitored using a confocal microscope (Zeiss LSM710, Germany).

### 2.5 Plasmid construction and transfection

The coding sequences of murine *Cdk9* were synthesized at Qi‐Long (Shanghai, China) and incorporated into the vector of pcDNA3.1 (+) (Invitrogen) at sites of Nhe I and Hind III. To increase expression CDK9 in primary chondrocytes, cells were transfected with a plasmid carrying *Cdk9* by electroporation with an apparatus (NEPA21, NEPA GENE, Japan).

### 2.6 Cell viability assay

Chondrocytes were seeded at 5 × 10^3^ cells/well in a 96‐well plate and cultured for 6 h in a cell culture incubator. Different dosages of EXOs (1 × 10^8^/mL, 2.5 × 10^8^/mL, 5 × 10^8^/mL, 1 × 10^9^/mL, 2 × 10^9^/mL) were added to cells and incubated for 24 h and 48 h. In OA cell model experiments, chondrocytes were treated with IL‐1β (10 ng/mL), IL‐1β (10 ng/mL) + CSPC‐EXOs (1 × 10^9^ p/mL), or CSPC‐EXOs (1 × 10^9^ p/mL) alone for 12, 24 and 48 h. Cell viability was detected using a cell counting kit (Dojindo Molecular Technologies, Kumamoto, Japan; CK04) according to the manufacturer's instructions.

### 2.7 Cell proliferation assay

Cell proliferation was analysed using the 5‐ethynyl‐2′‐deoxyuridine (EdU) labeling kit (Ribobio; Guangzhou, China; C10310‐3). Cells were incubated with 50 μM EdU in culture medium for 2–3 h. After washing twice with PBS, cells were fixed in 4% paraformaldehyde for 30 min. Glycine (2 mg/mL) was added and incubated for 5 min to remove aldehydes. After washing with PBS, cells were incubated with the Apollo staining solution. After 30 min‐incubation, the staining solution was removed, and cells were treated with 0.5% Triton X‐100 for 10 min. The nuclei were stained with Hoechst 3342. Cell images were captured using a fluorescence microscope (Life Technology; Evos FL Auto). The percentage of EdU‐positive cells was analysed to evaluate cell proliferation.

### 2.8 Scratch wound healing assay

Rat primary chondrocytes were seeded at 5 × 10^5^ cells per well. After 6 h, the culture inserts were removed, and non‐adherent cells were washed out with PBS. Cells were incubated with EXOs (1 × 10^9^/mL) and photographed at 12 and 24 h after treatment. The number of migrated cells was manually counted under an optical microscope. Three fields were counted per well. The area was determined using the ImageJ software (National Institutes of Health, USA).

### 2.9 Monosodium iodoacetate‐induced rat OA model

Male Sprague–Dawley rats with body weight ranging from 200 to 220 g were used in this study. Rats were randomly divided into five groups (8 rats per group) as indicated in related figure legends. Monosodium iodoacetate (MIA; Sigma‐Aldrich) was dissolved in 0.9% saline. The left knee joint was injected with 3 mg MIA in 50 μL volume using a 30‐G needle under isoflurane inhalation anaesthesia.^16^ The sham‐operated group was injected with an equal volume of PBS. The CSPC‐EXOs group was given 100 μL of EXOs (1 × 10^9^ particles) per joint. The control group was injected with PBS under the same conditions. In subacute OA group, 1 week after MIA injection, rats were received EXO treatment once a week for 21 days. In chronic OA group, 3 weeks after MIA injection, rats were treated once a week for 35 days. Rats were euthanized by CO_2_ inhalation at 28 days (subacute OA model) or 56 days (chronic OA model) after induction, and their knee joints were dissected for further analysis.

### 2.10 Histological analysis and evaluation of cartilage repair

Tissues were fixed in 4% paraformaldehyde for 24 h and then decalcified in 10% EDTA (pH 7.4) for 30 days. After paraffin embedding, tissues were cut into 5‐μm‐thick sections. Medial and lateral intervals were used for tissue sectioning with a spacing of 200 μm. After deparaffinization in xylene, the sections were rehydrated using a graded ethanol series. Then, they were stained with Safranin O/Fast Green (Solarbio; G1371‐5), haematoxylin and eosin, and toluidine blue. Immunohistochemical staining of Aggrecan (Servicebio, Wuhan, China; GB11373), COL‐I (Servicebio, Wuhan, China; GB11022), and COL‐II (Servicebio, Wuhan, China; GB11021) were then performed according to a previous protocol.^17^

### 2.11 Quantitative real‐time PCR (qRT‐PCR)

Total RNA extraction reagent RNA isolator (Vazyme) was used to RNA extraction from cells. RNA samples were then transcribed into cDNA using a cDNA Synthesis Kit (Vazyme). Levels of mRNA were analysed with iQ5 Multicolor Real‐Time PCR Detection System (Bio‐Rad) with FASTSTART ESSENTIAL DNA GREEN MASTER (Roche). The mRNA levels were normalized to the expression of 18S rRNA. The primer sequences are as following:

**Table:** 

Genes	Forward primer (5′‐3′)	Reverse primer (5′‐3′)
*18S rRNA*	AGTCCCTGCCCTTTGTACACA	CGTTCCGAGGGCCTCACT
*Tgfa*	TGATGCACTGAAGGTTAATATG	AACAAGGTACAATACAACTGAG
*Cdc7*	GGGTCTTAGGTAGTTGAGAAA	CCACATTCACAGCAGTAAC
*Prkca*	GGAAGGAGTGAAGGTTGA	GCATCTAACAGAGCGAATC
*Hif1a*	TGCCTAGTATGTTAATTTGTTGA	CAAAGAGCAAGGGAATGAAA
*Myocd*	ACTGGGAGAGCAAAGAAG	GAGAACAGGAAGCAATACAC
*Vegfa*	CGGTACTTATTTAATAGCCCTTT	GAGAGATTGGAAACACAGATTT
*Gpnmb*	GGGATGGGAAGACAGTATT	CGCTCAGGTATGTTCAATG
*Mmp2*	TTGCTTTGTTTGCCCTTT	TGACTGGAGTTGCTTCTAC
*Rufy3*	GAGCATCCACGAGAATCT	CTTCCACAAGGTCACACT

### 2.12 Protein extraction and western blot analysis

Cells were lysed in ice‐cold buffer containing 25 mM Tris–HCl, pH 7.4, 100 mM NaF, 50 mM Na_4_P_2_O_7_, 10 mM Na_3_VO_4_, 10 mM EGTA, 10 mM EDTA, 1% NP‐40, 10 μg/mL leupeptin, 10 μg/mL aprotinin, 2 mM PMSF, and 20 nM okadaic acid.^18^ Exosomal proteins were extracted using exosomal protein lysis buffer (101Bio).^19^ After 15 min‐centrifugation (12,000 rpm, 4°C), the supernatants were transferred into new tubes. Protein concentration in each sample was analysed by a protein assay kit (Bio‐Rad). 5× LaemmLi buffer was added to each sample and then proteins were denatured by boiling at 100°C for 5 min. The procedures for western blot were described elsewhere.^20^ Briefly, after cooling to room temperature, the prepared protein samples were resolved by SDS‐PAGE and then transferred to polyvinylidene fluoride membranes. The membranes were incubated with blocking buffer (Roche) for 1 h at room temperature, and then primary antibodies including anti‐CD9 (Proteintech; 20597‐1‐AP), anti‐CD63 (Abcam; ab134045), anti‐Calnexin (Proteintech; 10427‐2‐AP), or anti‐CDK9 (Proteintech; 11705‐1‐AP) were added, and the incubation were performed overnight at 4°C. The membranes were washed three times in TBST, and then incubated with the second antibody for 1 h at room temperature. After 3‐time washing in TBST, the membranes were developed with a chemiluminescence assay system and exposed to Kodak exposure films.

### 2.13 Proteomic analysis

Proteomic analysis was performed at Gene Denovo (Guangzhou, China). Briefly, after protein extraction from EXOs, samples were subjected to protein denaturation, reduction, alkylation, tryptic digestion and peptide cleanup. The resulting peptide mixture was re‐dissolved in the buffer A (20 mM ammonium formate, pH 10.0), and then fractionated by high pH separation using an Ultimate 3000 system (ThermoFisher Scientific, MA, USA) connected to a reverse phase column (XBridge C18 column, 4.6 mm × 250 mm, 5 μm, Waters Corporation, MA, USA). High pH separation was carried out using a linear gradient, starting from 5% B to 45% B in 40 min (B: 20 mM ammonium formate in 80% ACN, pH 10.0, adjusted with ammonium hydroxide). The column was re‐equilibrated at the initial condition for 15 min. The column flow rate was maintained at 1 mL/min and the column temperature was maintained at 30°C. Ten fractions were collected and each fraction was dried in a vacuum concentrator for the next step.

The peptides were re‐dissolved in 30 μL solvent A (A: 0.1% formic acid in water) and analysed by on‐line nanospray LC–MS/MS on an Orbitrap Fusion Lumos coupled to EASY‐nLC 1200 system (ThermoFisher Scientific, MA, USA). 3 μL peptide sample was loaded onto the analytical column (Acclaim PepMap C18, 75 μm × 25 cm) and separated with a 120‐min gradient, from 5% to 35% C (C: 0.1% formic acid in CAN). The column flow rate was kept at 200 nL/min with the column temperature of 40°C. The mass spectrometer was run under data dependent acquisition mode, and automatically switched between MS and MS/MS mode. The parameters are: (1) MS: scan range (*m/z*) = 350–1200; resolution = 120,000; AGC target = 400,000; maximum injection time = 50 ms; Filter dynamic exclusion: exclusion duration = 30 s; (2) HCD‐MS/MS: resolution = 15,000; AGC target = 50,000; maximum injection time = 35 ms; collision energy = 32. Raw data were processed and analysed by Spectronaut X (Biognosys AF, Switzerland) with default settings to generate an initial target list.

### 2.14 RNA sequencing (RNA‐Seq)

Total RNA was extracted using Trizol reagent kit (Invitrogen, Carlsbad, CA, USA) according to the manufacturer's protocol. RNA quality was assessed on an Agilent 2100 Bioanalyzer (Agilent Technologies, Palo Alto, CA, USA) and checked using RNase free agarose gel electrophoresis. After total RNA was extracted, eukaryotic mRNA was enriched by Oligo (dT) beads. Then, the enriched mRNA was broken into short fragments using fragmentation buffer and reversely transcribed into cDNA using NEBNext Ultra RNA Library Prep Kit for Illumina sequencing (NEB #7775, New England Biolabs, Ipswich, MA, USA). Next, the cDNA fragments were purified with QiaQuick PCR extraction kit (Qiagen, Venlo, Netherlands), end repaired, poly (A) added, and ligated to Illumina sequencing adapters. The ligation reaction was purified with AMPure XP Beads (1.0×). The ligated fragments were subjected to size selection by agarose gel electrophoresis and PCR amplification. The resulting cDNA library was sequenced using an Illumina Novaseq6000 at Gene Denovo Biotechnology Co., Ltd (Guangzhou, China).

### 2.15 Statistical analysis

The data are presented as the mean values ± SEM from a minimum of three independent experiments. Statistical analysis was conducted using GraphPad Prism software (Version 9.0.0; San Diego, CA, USA). One‐way analysis of variance (ANOVA) with Bonferroni's post hoc test was employed for statistical analysis. *p* < 0.05 was considered statistically significant.

## 3 RESULTS

### 3.1 Preparation and characterization of CSPC‐EXOs

**FIGURE 1:**
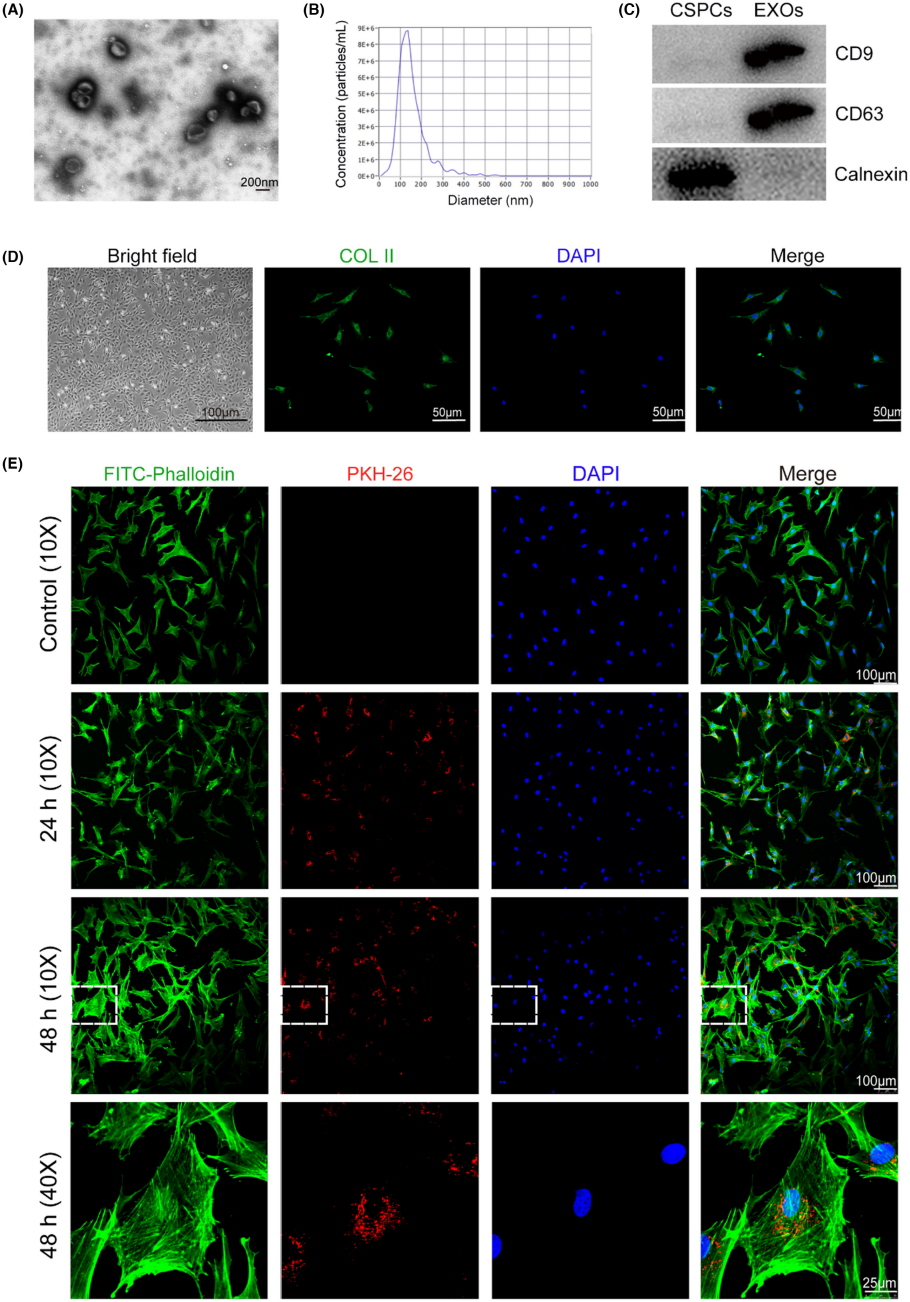
Preparation and characterization of cartilage stem/progenitor cells (CSPCs)‐derived EXOs. (A) Representative transmission electron microscopy (TEM) images of CSPC‐EXOs (scale bar = 200 nm). (B) Nanoparticle tracking analysis of CSPC‐EXOs. (C) Western blot analysis showing exosomal markers CD9 and CD63. Calnexin was used as a negative control. (D) Identification of rat primary chondrocytes. Cell morphology was observed with a light microscope (scale bar = 100 μm). Immunofluorescence analysis showing COL II (green) expression. DAPI (blue) was used to label the cell nuclei. Scale bar = 50 μm. (E) Internalization of CSPC‐EXOs in rat primary chondrocytes. CSPC‐EXOs were labelled with PKH26 (red) and co‐cultured with primary chondrocytes. FITC‐Phalloidin (green) and DAPI (blue) were used to label the cytoskeleton and the cell nuclei, respectively. Scale bar = 100 μm or 25 μm as indicated.

EXOs were prepared from CSPCs. Transmission electron microscope images showing CSPC‐EXOs are in a typical cup shape (Figure 1A). The average diameter of CSPC‐EXOs was 127.7 nm (Figure 1B). EXO protein markers, including CD9 and CD63, were present in CSPC‐EXOs, whereas Calnexin, a negative marker of EXOs, was not observed (Figure 1C). To examine the potential function of CSPC‐EXOs in cartilage repair, we first analysed their role in in vitro using chondrocyte cell culture. To this end, we prepared primary chondrocytes from newborn rats, that displayed a fusiform cell‐like morphology and were collagen II (COL II)‐positive (Figure 1D). Internalization tests showed that the CSPC‐EXOs we had prepared exhibited robust cell internalization ability in primary chondrocytes (Figure 1E). These data clearly indicate that the prepared extracellular vesicles are CSPC‐EXOs.

### 3.2 CSPC‐EXOs improve cell viability, migration, and proliferation in primary chondrocytes

**FIGURE 2:**
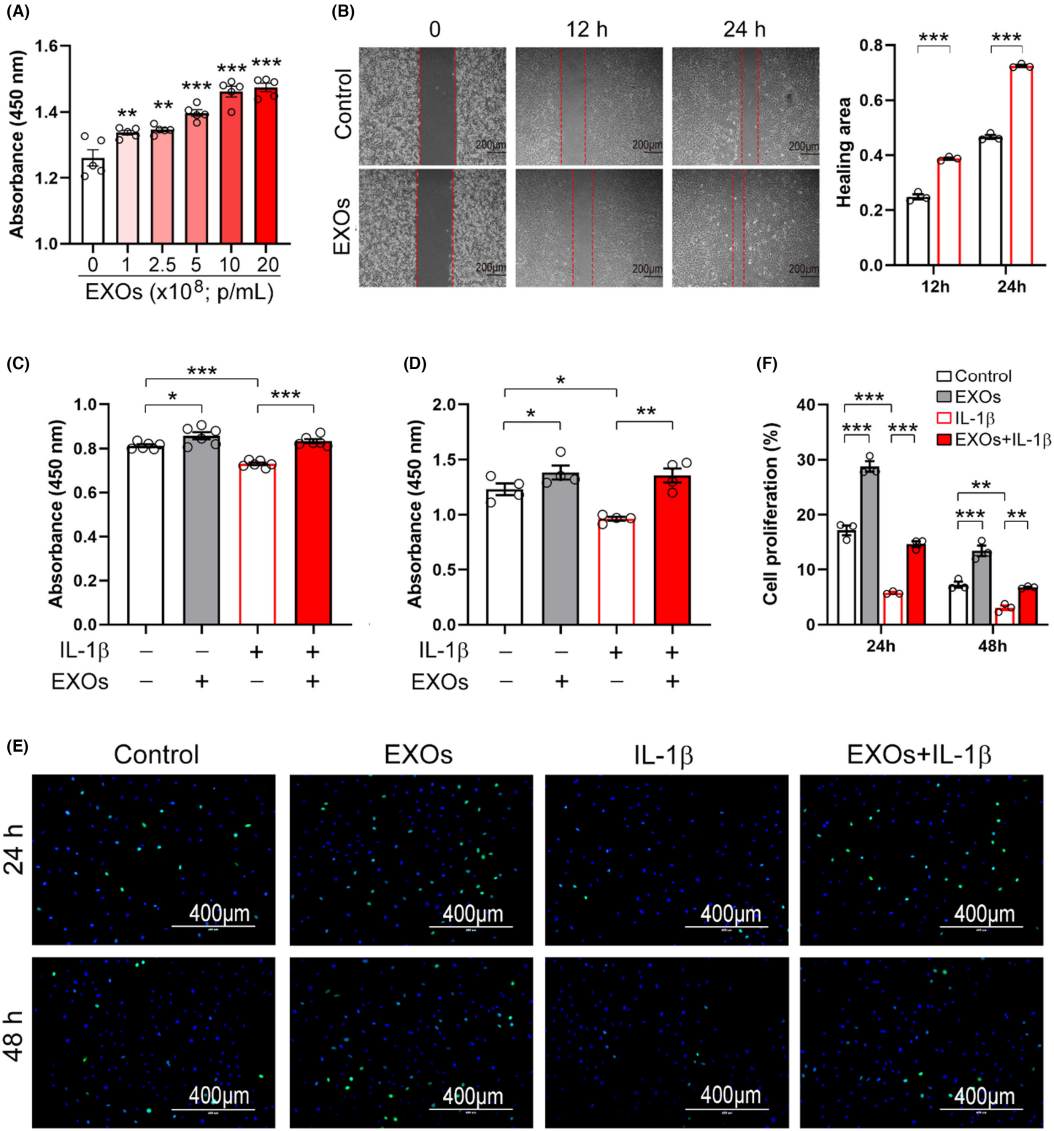
CSPC‐EXOs improve cell viability, migration, and proliferation in chondrocytes. (A) CSPC‐EXOs stimulate cell viability in primary chondrocytes. Cells were treated with EXOs at different dosages as indicated for 24 h, and then cell viability was analysed with a CCK‐8 kit. *n* = 5. (B) CSPC‐EXOs promote cell migration. Primary chondrocytes were treated with CSPC‐EXOs (1 × 10^9^ p/mL) for 12 h or 24 h, cell migration was analysed by wound healing assay. Scale bar = 200 μm. (C–D) CSPC‐EXOs counteract the decreased cell viability induced by IL‐1β in chondrocytes. Primary chondrocytes were treated with EXOs (1 × 10^9^ p/mL) and IL‐1β (10 ng/mL) as indicated for 12 h (C) and 24 h (D), cells were then subjected to cell viability assay with a CCK‐8 kit. *n* = 4–6. (E) CSPC‐EXOs improve cell proliferation in chondrocytes treated with IL‐1β. Primary chondrocytes were pretreated with CSPC‐EXOs at the concentrations of 1 × 10^9^ p/mL. 6 h post‐pretreatment, cells were incubated with 10 ng/mL of IL‐1β for 24 h and 48 h, EdU incorporation was used to analyse cell proliferation. Scale bar = 400 μm. (F) Quantification of cell proliferation as shown in (E). *n* = 3. Values are presented as mean ± SEM. *p < 0.05, **p < 0.01, ***p < 0.001, one‐way ANOVA.

We next examined whether CSPC‐EXOs affect cell growth and migration in chondrocytes. As shown in Figure 2A, EXOs increased the viability of primary chondrocytes in a dose‐dependent manner. Cell migration was also enhanced by EXOs (Figure 2B). Interleukin 1β (IL‐1β) treated chondrocytes are considered an OA cell model and were employed in experiments.^21^ Our results showed that cell viability was reduced in the presence of IL‐1β, while the application of EXOs mitigated this decline (Figure 2C,D). Moreover, the cell proliferation of primary chondrocytes was stimulated by EXOs, both in the absence or presence of IL‐1β (Figure 2E,F). These findings indicate that CSPC‐EXOs are capable of improving cell viability, migration and proliferation in chondrocytes, implying that CSPC‐EXOs may have therapeutic potential for treating cartilage defect‐associated diseases such as OA.

### 3.3 CSPC‐EXOs improve cartilage repair in OA model rats

**FIGURE 3:**
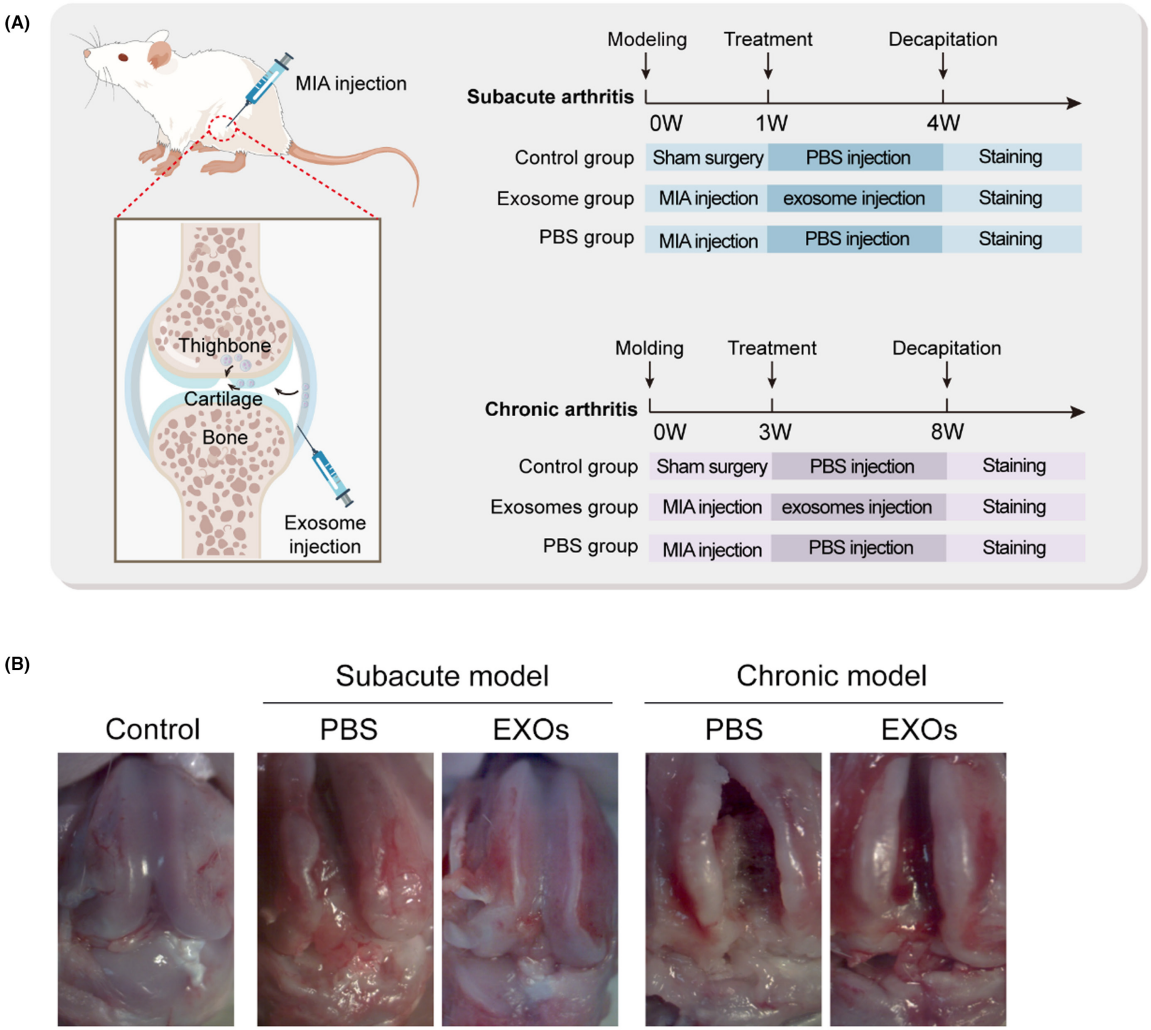
CSPC‐EXOs improve cartilage repair in subacute OA model rats. (A) Experimental timeline. (B) Macroscopic appearance of rat knee joints. MIA, monosodium iodoacetate.

**FIGURE 4:**
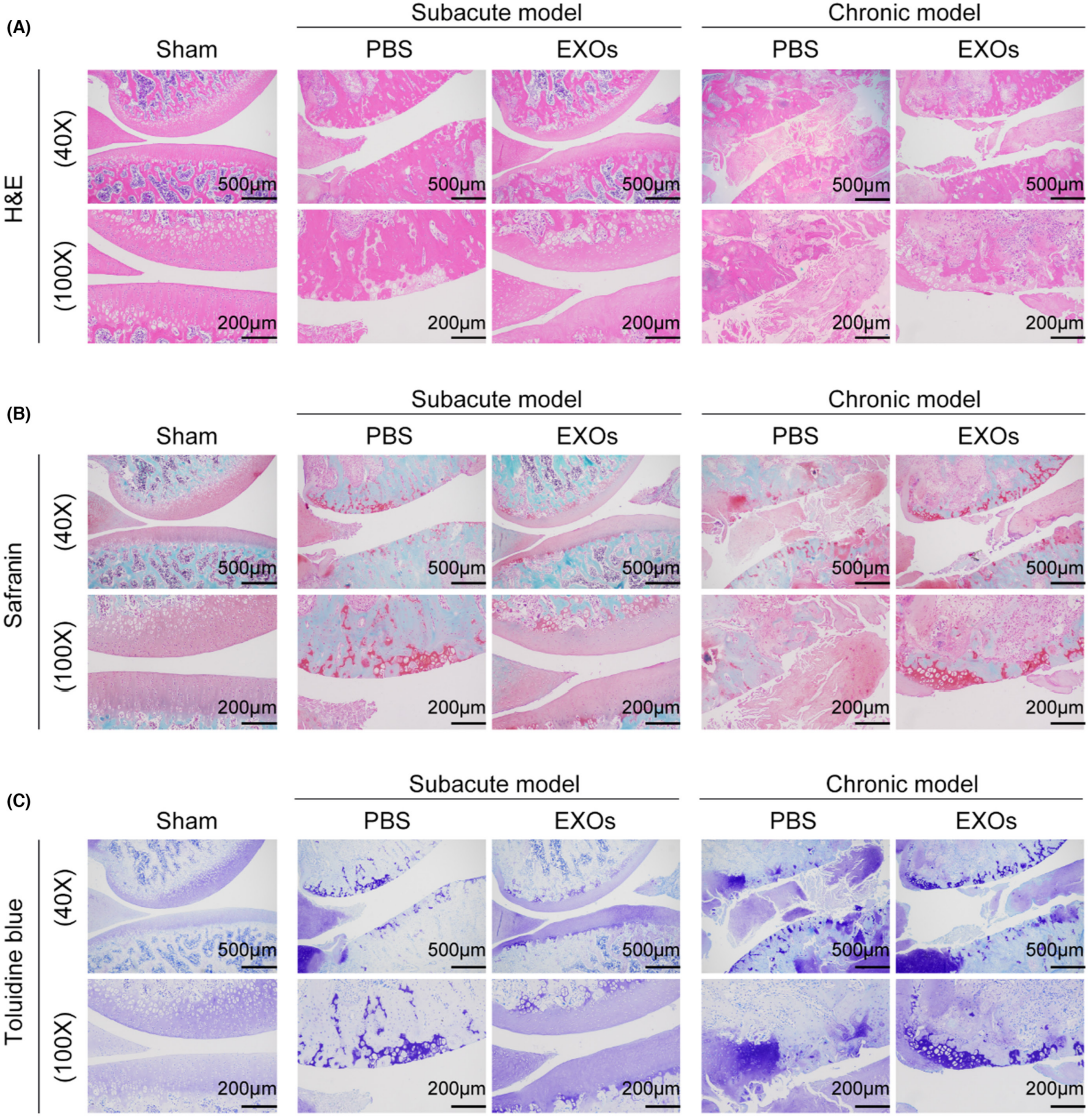
Representative images of haematoxylin and eosin (A), Safranin O/Fast Green (B) and Toluidine blue (C) staining of knee joint specimens. Scale bar = 500 μm (40×) or 200 μm (100×).

**FIGURE 5:**
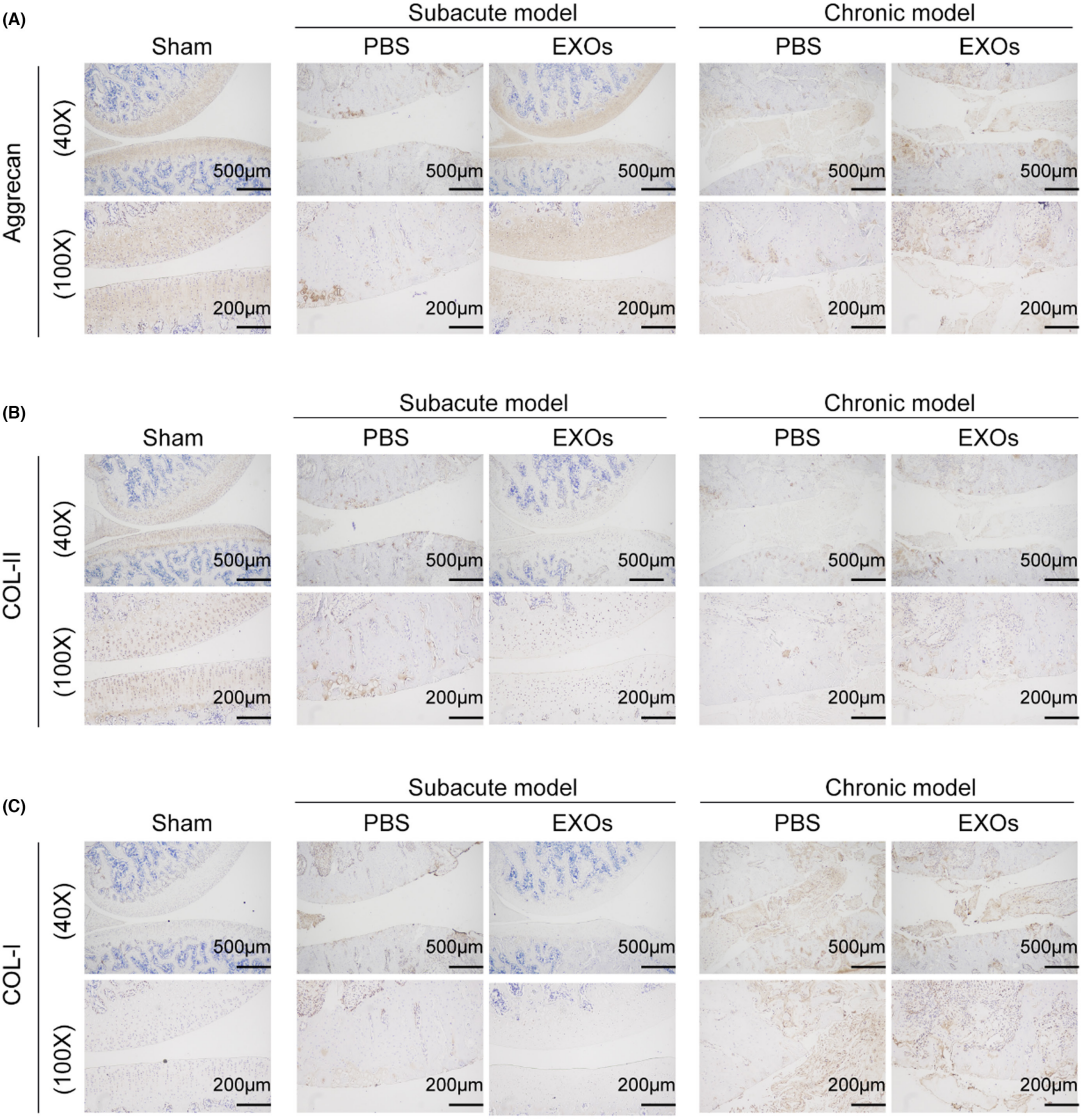
Immunohistochemical staining of Aggrecan (A), COL‐I (B) and COL‐II (C) in cartilage tissues. Scale bar = 500 μm (40×) or 200 μm (100×).

To evaluate the potential application of CSPC‐EXOs in cartilage repair, we generated an OA rat model by injecting MIA into the knee joint. The experimental timeline is shown in Figure 3A. At the end of the treatment, the rats were sacrificed and the knee joints were exposed. As shown in Figure 3B, a smooth and intact surface composed of cartilage was observed on the knee joint of the control group. In the OA rat model group of experimental animals, this structure was destroyed, as evidenced by a visible groove on the knee joint, especially in chronic OA model rats. However, the application of EXOs markedly attenuated this injury in subacute OA model rats (Figure 3B). In the chronic OA model rats, no significant changes were observed following EXOs treatment (Figure 3B). Histological analyses, including haematoxylin and eosin, Safranin and Toluidine blue staining, further confirmed that EXOs promoted cartilage regeneration in the knee joints of the subacute OA model rats (Figure 4A–C). Immunohistochemical staining also verified the benefits of EXOs on cartilage repair in subacute OA model rats, as evidenced by increased staining intensity for Aggrecan and COL‐II, and reduced expression of COL‐I (Figure 5A–C). In chronic OA model rats, EXOs treatment had no effect on cartilage regeneration but repressed fibrous granulation tissue accumulation in the joint space (Figures 4A–C and 5A–C). These data indicate that CSPC‐EXOs play a beneficial role in cartilage repair in an acute OA rat model.

### 3.4 Exosomal protein identification and bioinformatics analysis

**FIGURE 6:**
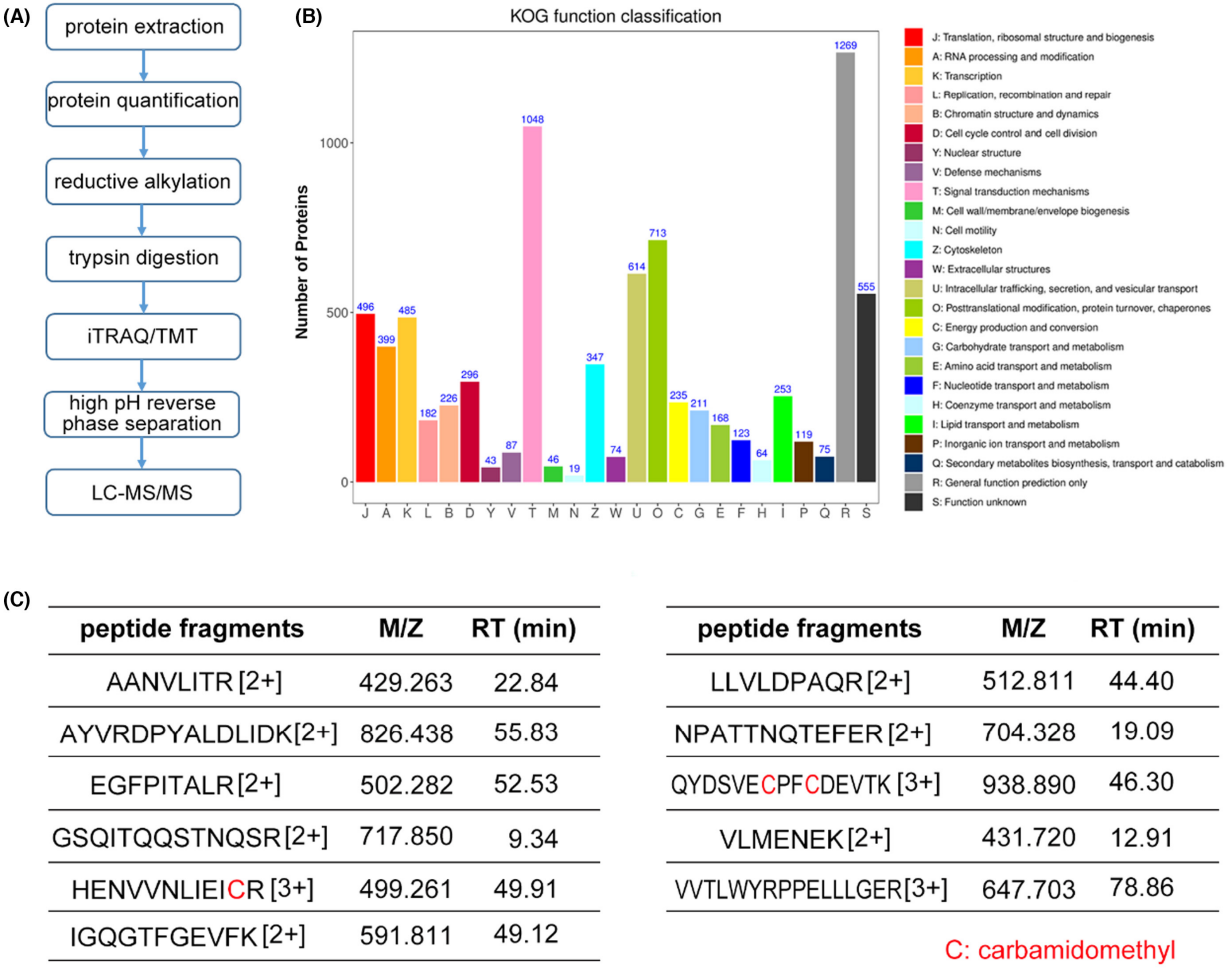
Exosomal protein identification. (A) The work flow of proteomic analysis. (B) The Eukaryotic Orthologous Group (KOG) functional classification. (C) The peptide fragments, m/z values, and retention time (RT) of CDK9 in mass spectrometry.

We aimed to examine the specific constituent(s) in CSPC‐EXOs that were responsible for improved cartilage repair. To this end, we performed proteomics analysis using liquid chromatography coupled with tandem mass spectrometry (LC–MS/MS) (Figure 6A). In total, 6777 proteins were identified in CSPC‐EXOs. The Eukaryotic Orthologous Group (KOG) functional classification showed that these proteins have multiple biological functions (Figure 6B). Among these annotations, module D attracted our attention because it comprises cell cycle and cell division, that are closely related to cell growth. There were 296 proteins in module D (Data **S1**), and among them CDK9 was considered because of its critical role in RNA polymerase II (RNAPII) transcription initiation, elongation, and termination,^22^ that are strongly correlated with cell growth. Mass spectrometry data showed the presence of 11 peptide fragments of CDK9, and this confirmed the presence of CDK9 in CSPC‐EXOs (Figure 6C).

### 3.5 Inhibition of CDK9 abolishes CSPC‐EXOs induced cell viability, migration, and proliferation in chondrocytes

**FIGURE 7:**
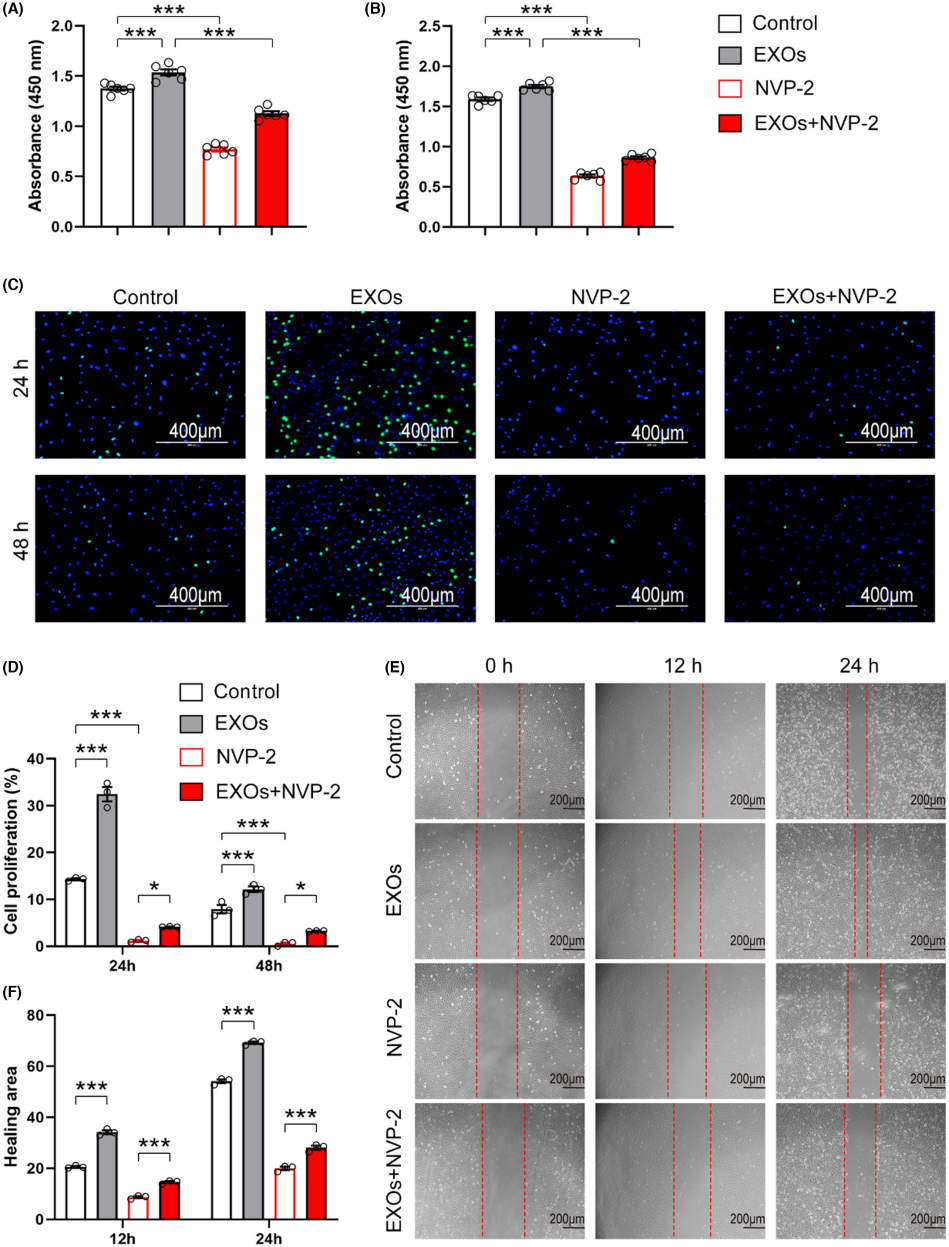
Inhibition of CDK9 diminishes CSPC‐EXOs induced cell viability, migration, and proliferation. (A–B) Inhibition of CDK9 by NVP‐2 mitigates the increased cell viability induced by CSPC‐EXOs. Primary chondrocytes were pretreated with NVP‐2 (2 μM) for 3 h, and then CSPC‐EXOs was added as indicated. After 24 h (A), and 48 h (B), cell viability was assayed with a CCK‐8 kit. *n* = 6. (C) NVP‐2 diminishes the enhanced cell proliferation induced by CSPC‐EXOs. Cell treatments were described in (A–B). Cell proliferation was analysed by EdU staining. Scale bar = 400 μm. (D) Quantification of cell proliferation as shown in (C). *n* = 3. (E) NVP‐2 counteracts CSPC‐EXOs induced cell migration. Cell treatments were described in (A–B). Cell migration was analysed by wound healing assay. (F) Quantification of healing area as shown in (E). *n* = 3. Values are presented as mean ± SEM. *p < 0.05, and ***p < 0.001, one‐way ANOVA.

To examine whether exosomal CDK9 plays a role in cell growth, we pretreated primary chondrocytes with NVP‐2, a potent inhibitor of CDK9, and incubated these cells with CSPC‐EXOs. As shown in Figure 7A,B, treatment with NVP‐2 markedly reduced cell viability. Notably, the increase in cell viability induced by EXOs was substantially prevented by NVP‐2 (Figure 7A,B). Similarly, the application of NVP‐2 repressed the proliferation and migration of primary chondrocytes in the presence or absence of EXOs (Figure 7C–F). These data suggest that exosomal CDK9 is a key factor in transducing the benefits of CSPC‐EXOs on cell growth in chondrocytes.

### 3.6 Overexpression of CDK9 recapitulates the benefits conferred by CSPC‐EXOs

**FIGURE 8:**
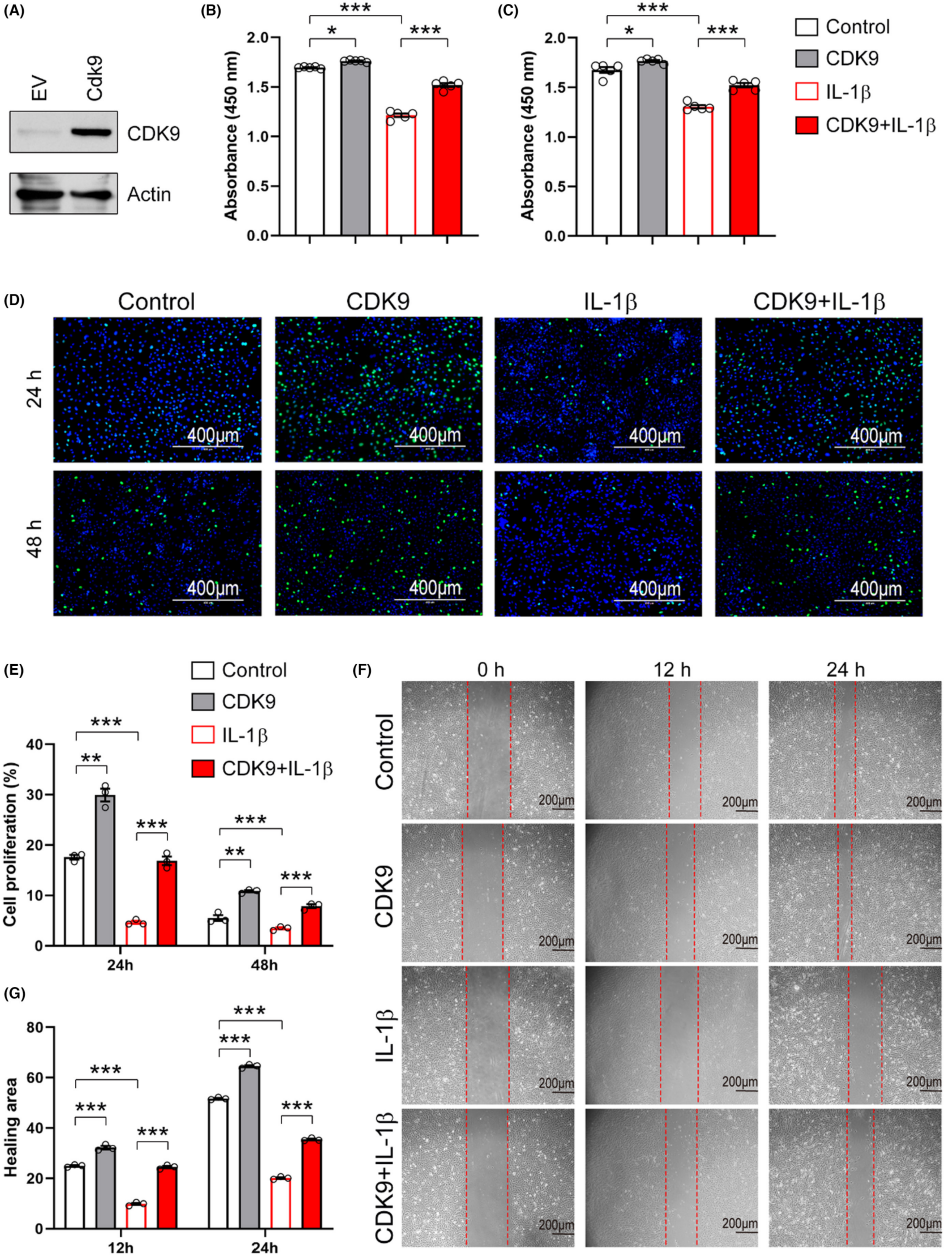
Overexpression of CDK9 prevents the reduced cell viability, migration, and proliferation in chondrocytes treated with IL‐1β. (A) Overexpression of CDK9 in chondrocytes. Primary chondrocytes were transfected with a plasmid expressing CDK9 by electroporation. 24 h post‐transfection, cells were harvested for western blot analysis. Actin was used as a loading control. EV, empty vector. (B–C) CDK9 improves cell viability. Primary chondrocytes were transfected with a plasmid expressing CDK9, 6 h post‐transfection, cells were treated with IL‐1β (10 ng/mL) for additional 24 h (B) and 48 h (C). Cell viability was examined with a CCK‐8 kit. *n* = 5. (D) CDK9 stimulates cell proliferation. Cell treatments were described in (B–C). Cell proliferation was assayed by EdU incorporation. Scale bar = 400 μm. (E) Quantification of cell proliferation as shown in (D). *n* = 3. (F) CDK9 induces cell migration. Cell treatments were described in (B–C). Cell migration was tested by wound healing assay. Scale bar = 200 μm. (G) Quantification of cell migration as shown in (F). *n* = 3. Values are presented as mean ± SEM. *p < 0.05, **p < 0.01, ***p < 0.001, one‐way ANOVA.

**FIGURE 9:**
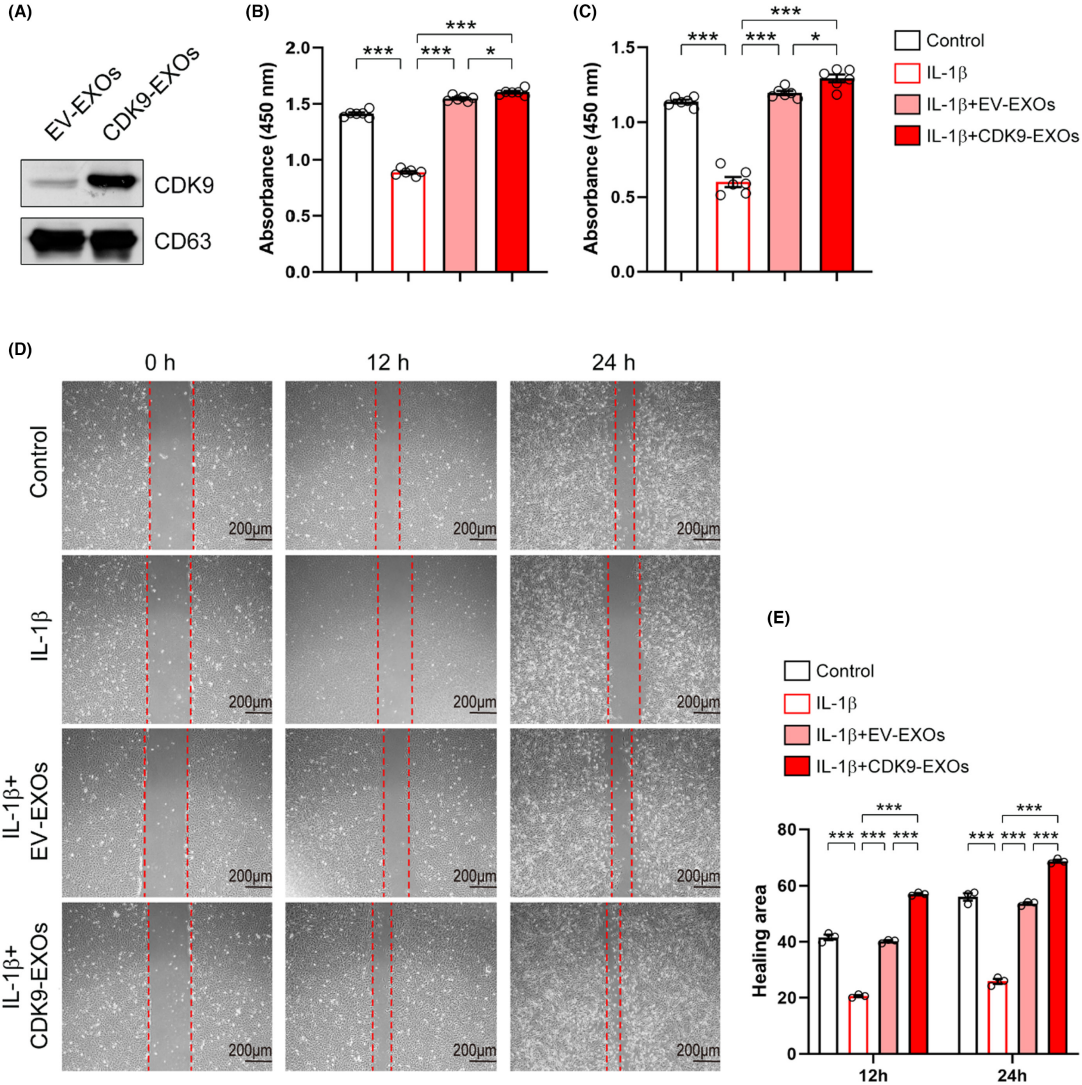
EXOs derived from CDK9‐overexpressed CSPCs have better performances in chondrocyte viability and migration. (A) Exosomal CDK9 was increased in EXOs derived from CDK9‐overexpressed CSPCs. EXOs were prepared from CSPCs transfected with a plasmid expressing *Cdk9* (CDK9‐EXOs) or empty vector (EV‐EXOs). CDK9 expression was analysed by western blot, and CD63 was used as a loading control. EV, empty vector. (B, C) CDK9‐EXOs increase the viability of chondrocytes. Primary chondrocytes were treated with IL‐1β (10 ng/mL), EV‐EXOs, and CDK9‐EXOs as indicated for 24 h (B) and 48 h (C). Cell viability was examined with a CCK‐8 kit. *n* = 6. (D) CDK9‐EXOs stimulates the migration of chondrocytes. Cell treatments were described in (B, C). Cell migration was tested by wound healing assay. Scale bar = 200 μm. (E) Quantification of cell migration as shown in (D). *n* = 3. Values are presented as mean ± SEM. *p < 0.05, ***p < 0.001, one‐way ANOVA.

To further confirm the critical role of CDK9 mentioned above, we constructed a recombinant plasmid expressing *Cdk9*, and this was then transfected into primary chondrocytes by electroporation. Western blot analysis showed that CDK9 expression was increased following transfection of the recombinant plasmid (Figure 8A). Similar to EXOs treatment, CDK9 increased the viability of primary chondrocytes in the absence or presence of IL‐1β (Figure 8B,C). Overexpression of CDK9 promoted EdU incorporation into newly synthesized genomic DNA in chondrocytes (Figure 8D,E). Moreover, CDK9 prevented the reduction in proliferation of IL‐1β‐treated cells (Figure 8D,E). Data from wound healing assay revealed that overexpression of CDK9 promoted cell migration in both control and IL‐1β‐treated chondrocytes (Figure 8F,G). To confirm the central role of exosomal CDK9 in chondrocyte growth, we prepared EXOs from CSPCs overexpressed CDK9 (CDK9‐EXOs; Figure 9A). As compared to EXOs derived from CSPCs transfected with empty vector (EV‐EXOs), CDK9‐EXOs exhibited better performances in the viability and migration of primary chondrocytes (Figure 9B–E). These data indicate that exosomal CDK9 plays a key role in inducing cell growth and migration in chondrocytes.

### 3.7 CSPC‐EXOs induce gene expression profiles in chondrocytes

**FIGURE 10:**
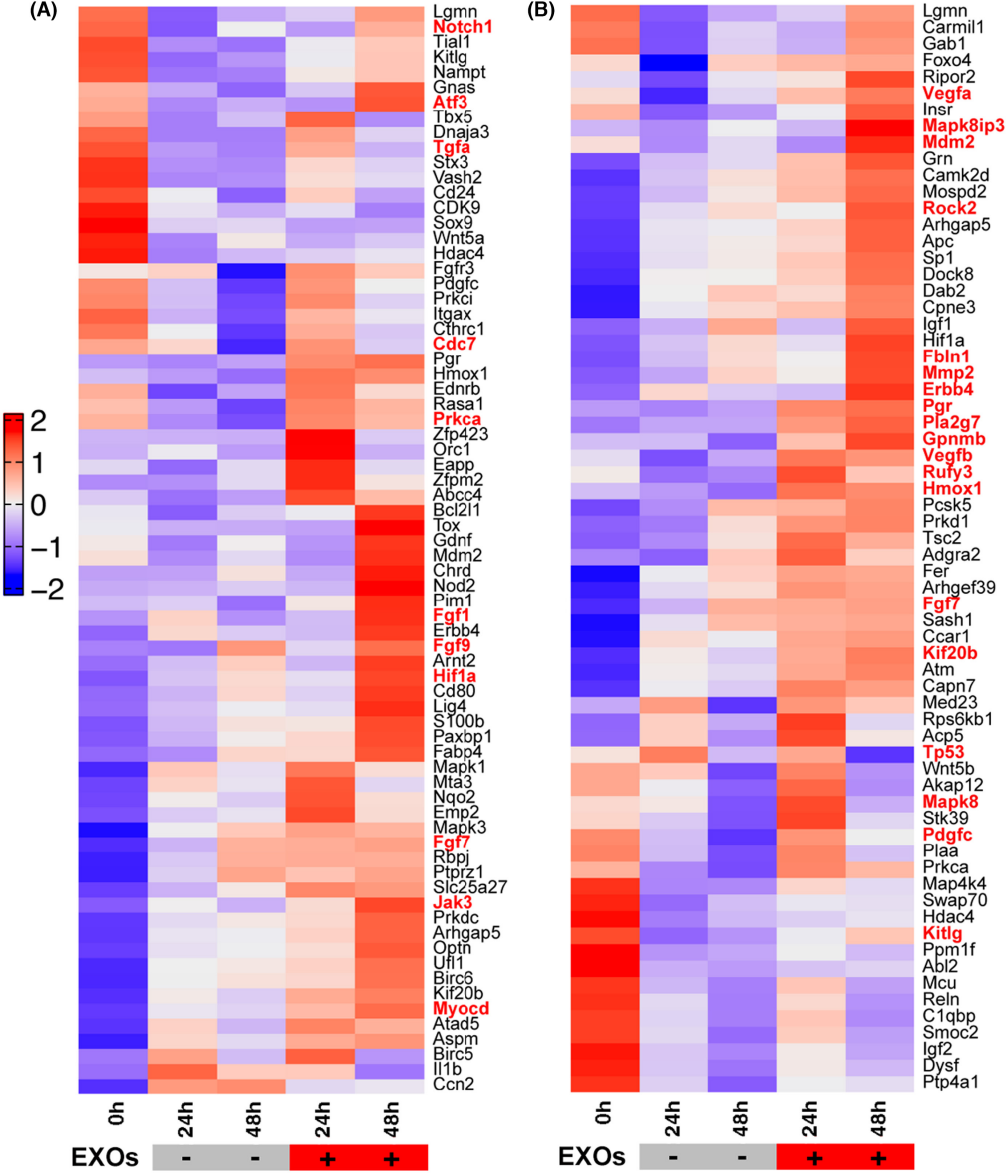
RNA‐Seq analysis showing differentially expressed genes induced by CSPC‐EXOs. Primary chondrocytes were treated with CSPC‐EXOs for 24 and 48 h, cells were harvested for RNA‐Seq. (A, B) The up‐regulated genes with potential role in cell proliferation (A) and cell migration (B).

**FIGURE 11:**
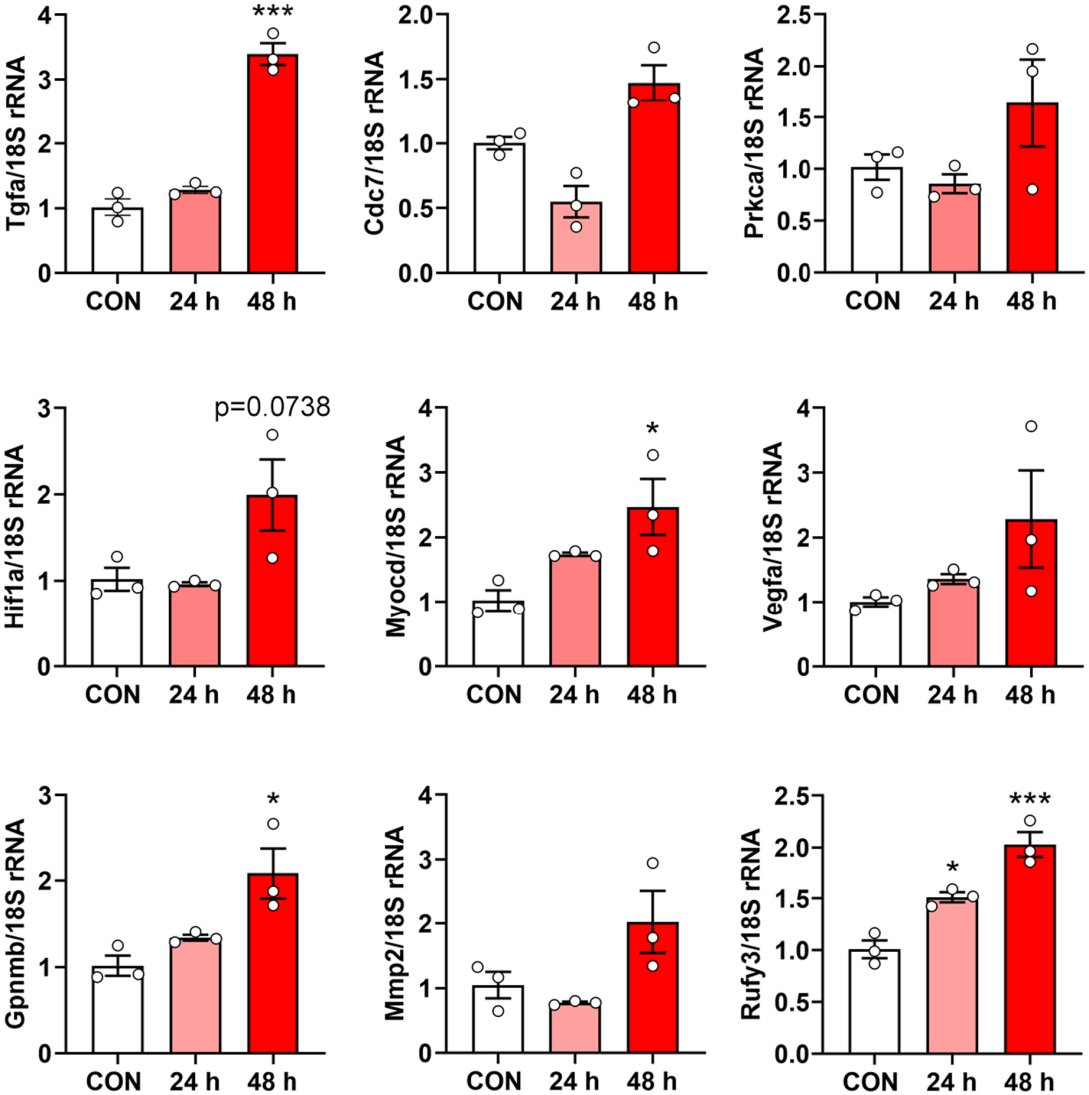
Effects of CDK9 on gene expression involved in cell proliferation and migration. Primary chondrocytes were transfected with the plasmid expressing CDK9 by electroporation. 24 and 48 h post‐transfection, cells were harvested for qPT‐PCR analysis, 18S rRNA was used as a house‐keeping gene. Values are presented as mean ± SEM. *n* = 3. *p < 0.05 and ***p < 0.001 versus CON, one‐way ANOVA.

In the following experiments, we analysed the gene expression profiles induced by CSPC‐EXOs in chondrocytes. To this end, we performed RNA‐Seq analysis in primary chondrocytes treated with or without CSPC‐EXOs. Due to the increased cell proliferation and migration in EXOs‐treated chondrocytes, we focused on genes involved in these two cell processes. Based on bioinformatics analysis, the differentially expressed genes with potential roles in cell proliferation and migration were plotted in heatmaps. The differentially expressed genes that correlate with cell proliferation are shown in Figure 10A, and include *Notch1*, *Atf3*, *Tgfa*, *Cdc7*, *Fgf1*, *Fgf9*, *Hif1a*, *Jak3* and *Myocd*. The differentially expressed genes correlating with cell migration are shown in Figure 10B, and include *Vegfa*, *Mapk8ip3*, *Rock2*, *Fbln1*, *Mmp2*, *Erbb4*, *Pgr*, *Pla2g7*, *Gpnmb*, *Vegfb*, *Rufy3*, *Hmox1* and *Fgf7*. These data reveal the underlying mechanisms of EXO‐induced cell proliferation and migration in chondrocytes. To reconcile the key role of CDK9 in cell proliferation and migration, we transfected primary chondrocytes with a plasmid bearing *Cdk9* by electroporation to mimic the application of EXOs, and then analysed whether these differentially expressed genes exhibited expression patterns similar to those induced by EXOs. As shown in Figure 11, among the nine genes whose expression patterns were tested, four genes namely *Tgfa*, *Myocd*, *Gpnmb* and *Rufy3*, were stimulated by CDK9 at 24 and 48 h post‐transfection. These findings reveal the underlying mechanism for CSPC‐EXOs induced cell growth and migration in chondrocytes, and show that, exosomal CDK9 plays a pivotal role.

## 4 DISCUSSION

In this study, we prepared EXOs from primary cartilage progenitor/stem cells and examined their potential applications in the treatment of cartilage degeneration related diseases such as OA. Our data showed that CSPC‐EXOs play a role in the viability, proliferation, and migration of primary chondrocytes. In OA model rats, the application of CSPC‐EXOs promoted cartilage repair in the knee joint. Proteomics and bioinformatics analyses, and functional studies revealed that CDK9 present in CSPC‐EXOs is a key factor in inducing cell growth and migration.

CSPCs possess a therapeutic potential comparable to other stem cell types and have a more robust capacity for chondrogenic differentiation than other mesenchymal cells such as stem/progenitor cells from human adipose or bone marrow.^23^ Additionally, their osteogenic differentiation potential remains limited, making them ideal candidates for cartilage tissue engineering applications.^24^, ^25^, ^26^, ^27^ Therefore, CSPC‐based cell therapy has great potential for the treatment of cartilage degeneration. However, stem cell‐based therapies have several shortcomings, including limited stem cell resources, poor cell viability after implantation, uncontrolled differentiation in vivo, and immune rejection in allografts.^28^ Recently, an increasing number of studies have shown that stem cell extracellular vesicles exhibit similar functions without these shortcomings.^17^, ^29^ Therefore, stem cell extracellular vesicle‐based therapy is of great importance in tissue engineering and translational medicine.^30^, ^31^, ^32^

EXOs, a subpopulation of extracellular vesicles, are small membranous vesicles secreted by various cell types that range from 40 to 160 nm in diameter.^12^ EXOs have been shown to play a crucial role in intercellular communication by transferring bioactive molecules including proteins, nucleic acids and lipids between cells.^12^ These nanoscale vesicles possess inherent biocompatibility, stability and the ability to traverse biological barriers.^33^ Studies have demonstrated the therapeutic potential of MSC‐derived EXOs in cartilage regeneration. For instance, small extracellular vesicles from human adipose‐derived stem cells exhibited significant attenuation of OA progression and preservation of cartilage integrity against degeneration in a murine MIA model.^16^ Another study showed that EXOs derived from synovial fluid MSCs facilitate chondrogenesis and induce cartilage regeneration.^34^ Additionally, EXOs derived from human MSCs have been found to promote cartilage regeneration in rats, rabbits and micropigs.^17^, ^35^, ^36^ In line with these findings, in the present study, we observed that EXOs derived from CSPC favoured cartilage repair in a subacute OA rat model.

It has been shown that exosomal proteins play a key role in transducing the benefits conferred by EXOs in various disease models. For example, exosomal HSP72 triggers myeloid‐derived suppressor cell activation via a STAT3‐dependent immunosuppressive function.^37^ Gastric cancer cell‐derived exosomal EGFR promotes liver‐specific metastasis in Kupffer cells and hepatic stellate cells by enhancing HGF signalling in the liver.^38^ Most recently, fibroblast exosomal TFAP2C was shown to promote peripheral axon regeneration via the miR‐132‐5p/CAMKK1 axis.^19^ Therefore, in this study, we analysed exosomal proteins using proteomics to identity the proteins that play a key role in chondrocyte growth. Using bioinformatics, CDK9 was selected as a potential key factor for transducing the benefits of CSPC‐EXOs in cartilage repair. Our functional studies reported here confirmed this prediction: overexpression of CDK9 increased cell viability, migration and proliferation in chondrocytes, whereas inhibition of CDK9 induced opposite phenotypes. As a transcription hub, CDK9 plays an essential role in efficient transcription of most RNAPII‐transcribed genes.^22^ Hence, it is reasonable to predict that CDK9 regulates the expression of cell growth‐ and migration‐related genes in chondrocytes. It is well documented that CDK9 is a potent activator of cell proliferation, and inhibition of CDK9 impedes cell growth and proliferation.^39^, ^40^, ^41^ These previous results are consistent with our present findings in chondrocytes. However, the mechanism underlying CDK9‐induced cell growth and migration in chondrocytes remains unknown. Owing to the essential role of CDK9 in transcription,^22^ large‐scale gene expression profiling appears to be an ideal approach for addressing this issue. We performed RNA‐Seq analysis in chondrocytes treated with CSPC‐EXOs. Our results showed that several genes involved in cell growth and migration were upregulated. Of these, nine genes were selected to verify the underlying mechanism of action of CDK9, and we found that four genes were stimulated by CDK9 in chondrocytes. These results indicate that CDK9 alone cannot fully recapitulate the outcomes induced by CSPC‐EXOs, but plays a key role in chondrocyte growth and migration.

## 5 CONCLUSION

In conclusion, CSPC‐EXOs promoted the viability, migration, and proliferation of primary chondrocytes. In a rat model of subacute OA, the application of CSPC‐EXOs improved cartilage repair in the knee joint. Proteomics and bioinformatics analyses suggested that CDK9 is a key factor in CSPC‐EXO‐induced cell growth and migration in chondrocytes. Our functional studies confirmed the prediction that CDK9 derived from CSPC‐EXOs induces cell growth and migration in chondrocytes. Together, our data indicate that CSPC‐EXOs may represent a cell‐free therapy option for treating cartilage defect‐associated diseases such as OA. Moreover, we speculate that CDK9 may be a drug target for developing agents with therapeutic potential for cartilage regeneration.

## AUTHOR CONTRIBUTIONS

**Jing Chen:** Formal analysis (lead); investigation (lead); visualization (equal); writing – original draft (equal). **Xiaohui Ni:** Methodology (equal); validation (equal). **Jian Yang:** Formal analysis (supporting); methodology (equal). **Hongwei Yang:** Methodology (lead). **Xiaoyu Liu:** Resources (lead). **Minhao Chen:** Formal analysis (equal). **Cheng Sun:** Conceptualization (equal); funding acquisition (lead); project administration (lead); supervision (equal); writing – review and editing (equal). **Youhua Wang:** Conceptualization (equal); funding acquisition (equal); project administration (equal); supervision (equal).

## FUNDING INFORMATION

This work was supported by the National Natural Science Foundation of China (32271193); the Jiangsu Provincial Medical Key Discipline (Laboratory) Cultivation Unit (JSDW202205); and the Project Funded by the Priority Academic Program Development of Jiangsu Higher Education Institutions (PAPD).

## CONFLICT OF INTEREST STATEMENT

The authors have no conflicts of interest to declare.

## Supporting information


Data S1.

## Data Availability

All presented data were including in this published article. RNA‐seq data are available at http://db.cngb.org/cnsa/project/CNP0005024_995fadd9/reviewlink/.
